# The effect of omega‑3 fatty acid supplementation on clinical and biochemical parameters of critically ill patients with COVID‑19: a randomized clinical trial

**DOI:** 10.1186/s12967-022-03237-6

**Published:** 2022-01-15

**Authors:** Seyedeh Zoha Safaei Ardestani, Seyedeh Tayebeh Rahideh

**Affiliations:** Department of Nutrition, School of Public Health, Iran University of Medical Sciences, Tehran, Iran

Dear Editor,

We read with great interest the article “The effect of omega‑3 fatty acid supplementation on clinical and biochemical parameters of critically ill patients with COVID‑19: a randomized clinical trial” by Doaei et al. [1]. This study has an interesting idea, but we think there are some issues in the study conduct and interpretation that should be considered or need more explanation.

The authors offered no explanations for choosing the daily dose of 1000 mg omega-3 being received in the intervention group, nor did they clarify why the study period of two weeks was considered.

In this study, the authors explained: “all participants received high protein formula as 30 kcal/kg/d” but data for the actual calorie intake in the two groups were not provided. Furthermore, it is reported that the control group received isocaloric-isovolemic formula yet the caloric intake and *p*-value between the two groups are not presented. In other words, it was better to report p-value of calorie intake between the two groups, because a difference in caloric intake between the groups might influence the findings.

The used reference and formula for calculating the sample size are missing. Besides, it would have been better if the authors clearly stated in the abstract that 128 people enrolled, but the final analysis was done on 101 people.

As regards the loss to follow-up bias that has occurred, the marginal sample size should have been reported.

According to the baseline data in the table-1 of the article, the groups were significantly different in terms of one-month survival rate and urine volume mL/day. This matter is not only highlighted in the body text but also failed to take into account for result adjustment. For instance, it was important to adjust the one-month survival rate. In our opinion this observation may not have been due to the effect of the intervention because there was a significant difference between the two groups at baseline. Since the excreted urine amount in the intervention group was claimed to be marginally significant higher than the control group after the intervention, it was better to consider adjusting for the baseline urine output.

To more accurately assess the effects of omega-3 supplements, omega-3 baseline levels should be examined and the omega-3 fatty acid status in participants should have been investigated.

Examination of omega-3 supplementation effect on inflammatory markers is represented as one of the main objectives of the study; however, the authors fail to measure some relevant outcomes variables of the study protocol, especially those associated with inflammatory markers such as LDH and CRP [2].

The study would have been far more useful if authors had assessed Erythrocyte sedimentation rate (ESR). Which are good indicators for disease severity prediction in SARS-CoV-2 infected patients [3].

At last, this study did not specify the drugs used in the intervention group as well as their doses to investigate drug interactions. For instance, aspirin, which can have beneficial effects in patients with COVID-19 [4], was most likely taken by the participants. In the presence of aspirin EPA and DHA are converted to potent inflammation resolving mediators like resolvins. Thus it would be useful to know the medications used in both groups.

## Data Availability

Not applicable.
